# Functional localization of audiovisual speech using near infrared spectroscopy

**DOI:** 10.1007/s10548-022-00904-1

**Published:** 2022-07-12

**Authors:** Iliza M. Butera, Eric D. Larson, Andrea J. DeFreese, Adrian KC Lee, René H. Gifford, Mark T. Wallace

**Affiliations:** 1 Vanderbilt Brain Institute, Vanderbilt University, Nashville, TN USA; 2 Institute for Learning & Brain Sciences, University of Washington, Seattle Washington, USA; 3 Department of Hearing and Speech Sciences, Vanderbilt University, Nashville, TN USA; 4 Department of Speech and Hearing Sciences, University of Washington, Seattle, Washington USA; 5 Vanderbilt Kennedy Center, Vanderbilt University Medical Center, Nashville, TN USA

**Keywords:** Multisensory integration, fNIRS, Infrared spectroscopy, Speech in noise, McGurk effect

## Abstract

Visual cues are especially vital for hearing impaired individuals such as cochlear implant (CI) users to understand speech in noise. Functional Near Infrared Spectroscopy (fNIRS) is a light-based imaging technology that is ideally suited for measuring the brain activity of CI users due to its compatibility with both the ferromagnetic and electrical components of these implants. In a preliminary step toward better elucidating the behavioral and neural correlates of audiovisual (AV) speech integration in CI users, we designed a speech-in-noise task and measured the extent to which 24 normal hearing individuals could integrate the audio of spoken monosyllabic words with the corresponding visual signals of a female speaker. In our behavioral task, we found that audiovisual pairings provided average improvements of 103% and 197% over auditory-alone listening conditions in −6 and −9 dB signal-to-noise ratios consisting of multi-talker background noise. In an fNIRS task using similar stimuli, we measured activity during auditory-only listening, visual-only lipreading, and AV listening conditions. We identified cortical activity in all three conditions over regions of middle and superior temporal cortex typically associated with speech processing and audiovisual integration. In addition, three channels active during the lipreading condition showed uncorrected correlations associated with behavioral measures of audiovisual gain as well as with the McGurk effect. Further work focusing primarily on the regions of interest identified in this study could test how AV speech integration may differ for CI users who rely on this mechanism for daily communication.

**Supplementary Information:**

The online version contains supplementary material available at 10.1007/s10548-022-00904-1.

## Introduction

When auditory and visual stimuli originate from the same source at approximately the same time, the viewer may perceptually “bind” them into a single, audiovisual percept. This process of cue combination is called multisensory integration and it occurs on a daily—if not constant—basis to filter and combine sensory inputs into coherent percepts. Integration is determined by the temporal and spatial congruity of sensory inputs, and the perceptual benefits of their integration is increasingly apparent when the components are relatively weak (e.g., quiet) or otherwise obscured (e.g., embedded in noise).

Oral communication is inherently multisensory, and the addition of background noise makes it readily apparent how visual articulations contribute to speech understanding. The “cocktail party effect” describes the common circumstance in which attention must be focused on a single conversation while in the presence of many others. Both informational and energetic masking can be significantly reduced or even eliminated via lipreading (Ross et al. 2007), and these perceptual benefits have been known for some time (Sumby and Pollack 1954).

Complementary visual information not only effectively enhances the signal-to-noise ratio (SNR) for speech perception in noise, but has also been shown to alter the content of audible speech, as illustrated by the McGurk effect. In this audiovisual illusion, incongruent auditory and visual speech tokens are combined into a novel, fused percept (McGurk and McDonald, 1976). There are many methods for measuring the integration of auditory and visual information, and the McGurk effect is perhaps the one most extensively studied (Stevenson et al. 2014a). Several different stimulus combinations can elicit the illusion (e.g., visual “ga” and auditory “ba” to elicit an illusory “da” or “tha”), all of which follow the pattern of pairing a viseme with low visual saliency (i.e., velar articulation at the back of the tongue) with an auditory phoneme that would typically have a clear bilabial articulation. In fact, a sound like “ba” has such unambiguous articulation that it can even be identified via lipreading with a higher accuracy than auditory-only listening (Butera et al. In Press). Thus, pairing stimuli with both highly contrasted visual sensory estimates and incongruent places of articulation leads to their multisensory fusion into an alveolar intermediate. Many studies have investigated the different unisensory components that cause this illusion (Rouger et al. 2008), its emergence in infancy (Kushnerenko et al. 2008) and early childhood development (Tremblay et al. 2007; Hirst et al. 2018), changes with hearing loss (Desai et al. 2008; Rosemann et al. 2020) and aging (Cienkowski and Carney 2002), as well as the stimulus dependency of the illusion (Mallick et al. 2015) that computational models can better mitigate (Magnotti and Beauchamp 2015; Stropahl et al. 2017).

While the McGurk illusion is well-characterized as a measure of audiovisual integration, and there are known impairments in several neurological disorders (Pearl et al. 2009; Woynaroski et al. 2013; Stevenson et al. 2014b), this and other tests of audiovisual function are not typically used in clinical settings. Hearing and vision are clearly interrelated for successful speech understanding; however, their assessments and treatments remain largely segregated to separate clinics and specialists. In audiology, for instance, vision is well-known to have an important role in communication and validated measures for assessing AV integration are available (e.g., Holt et al., 2011), though these materials are rarely, if ever, used in clinical test batteries. Audiovisual tests are useful as more ecologically-valid estimates of speech intelligibility, and the importance of concurrent visual speech cues is typically amplified in those with hearing loss.

One clinical population in which AV integration is likely to play an important role is cochlear implant (CI) users. Following onset of severe-to-profound sensorineural hearing loss, these individuals have undergone the surgical placement of an electrode array inside the cochlea in order to electrically excite surviving spiral ganglion cells and, thus, regain auditory perception. Although the electrical pulses delivered through a CI are only a crude representation of the spectrally and temporally complex auditory signals that make up speech and other environmental sounds, successful CI surgery can result in remarkable gains in speech comprehension (Blamey et al. 2013a). Deciphering auditory information is at the core of CI technology, but visual information is likely to also play an important role in implant success—particularly for speech recognition in noisy environments. In what is typically an easy and common scenario for normal hearing listeners, average sentence recognition at a + 5 dB SNR is in the range of 30–50% correct for postlingually deafened adult CI users [e.g., (Gifford et al. 2018; Dunn et al. 2020)]. This reduction in auditory saliency suggests that audiovisual integration may be a common compensatory strategy for CI users. Furthermore, plasticity associated with hearing loss and its subsequent restoration is not solely limited to the auditory modality, and likely also entails changes to visual and audiovisual brain regions such that mechanisms of integration may differ as well (Merabet and Pascual-Leone 2010).

The superior temporal sulcus (STS) is a brain region known to be involved in the integration of AV speech, and the organization of auditory and visual inputs to the STS are well characterized (Beauchamp et al. 2004). However, limitations in available imaging techniques have been a barrier for mapping these inputs and their active integration in CI users. Like fMRI, functional near infrared spectroscopy (fNIRS) is used for noninvasive functional imaging of hemodynamic responses in the brain, yet without potential CI-related safety concerns of shifting electrodes, demagnetizing implants, and excessively heating surrounding tissues during MRI (Majdani et al. 2008). Instead, the optical basis of fNIRS utilizes low energy light emitters tuned to wavelengths in the near infrared spectrum (typically within the range of ~ 650–900 nm). Using photodiode detectors, recordings are made of the changes in light absorption that result from fluctuating concentrations of oxygenated and deoxygenated hemoglobin in proximal tissues. This serves as a blood-oxygen-level dependent (BOLD) signal from which underlying physiological processes can be inferred. Unlike the indirect measure of disturbances in a magnetic field by these compounds in MR-derived BOLD signals, NIRS is a direct measure of their concentrations at a much higher sampling rate. In addition, fNIRS has several other unique advantages including: quiet operation, resistance to movement artifacts, insensitivity to electrical interference, and for certain systems, portability.

The major limitation of fNIRS revolves around spatial resolution. Infrared light can travel approximately 1-2 cm below the scalp before scattering, which corresponds to around 5-15 mm of cortex (Strangman et al. 2014; Scholkmann et al. 2014). Fortunately, prior studies have indicated a high correspondence between an fMRI-derived BOLD signal and fNIRS (Ferradal et al. 2014), including marked similarities in the hierarchical speech processing involved in auditory sentence comprehension as measured by both fMRI and fNIRS (Hassanpour et al. 2015).

Neural mechanisms underlying the McGurk illusion have been measured via a range of neuroimaging techniques including fMRI (Nath et al. 2011; Nath and Beauchamp 2012), EEG (Saint-Amour et al. 2007; Kushnerenko et al. 2008; Shahin et al. 2018), TMS (Beauchamp et al. 2010), and a large-scale lesion study of stroke patients (Hickok et al. 2018). There is much support in the literature (from these and many other studies) that the STS is a key locus in the McGurk illusion. While there is a growing body of fNIRS literature assessing speech and language in CI users [for a review, see (Bortfeld 2019)], there is only one fNIRS study of the McGurk effect in infants with normal hearing (Ujiie et al. 2020) and, to our knowledge, none in either CI users or normal hearing adults.

### Purpose of the current study

Auditory deprivation and cochlear implantation are known to cause compensatory brain plasticity, which has prompted the fields of auditory neuroscience and clinical audiology to better characterize auditory, visual, and AV function along with their neural correlates in listeners with hearing loss. In the current work, we aimed to characterize behavioral performance and optically-derived hemodynamic responses to speech-in-noise stimuli in NH controls in order to establish an important baseline for future comparisons with CI users via an implant-compatible neuroimaging technique.

Further context for this area of research is based in the development of evidence-based interventions for auditory- and audiovisual-based rehabilitation, which must be founded in the neural processes underlying AV speech recognition in noise. This is an important consideration given the clinical promotion of auditory training that discourages the use of visual cues for communication in listeners with hearing loss [i.e., auditory-verbal therapy (AVT)]. Thus, the primary goal of the current study is to design and test behavioral tasks of AV integration in various SNRs yielding CI-level auditory performance, and relate this behavioral metric to neuroimaging measures of brain activity using fNIRS. In addition, we sought to compare both unisensory (i.e., auditory alone, visual alone) and multisensory (i.e., AV) performance on the McGurk effect, an illusion that represents a well-described proxy measure for the strength of integration of auditory and visual speech tokens.

## Materials and methods

**Participants.** We recruited 24 adults with normal hearing to participate in a two-hour study of behavioral speech testing and optical neuroimaging. All participants were native English speakers and passed bilateral hearing screening such that auditory detection levels were ≤ 20 dB HL from 250 to 4000 Hz, in octave steps. The average age was 25.4 years (SD = 5.1), and 45% were female (n = 11). Twenty-one participants (88%) were right-handed. All procedures were approved by the Vanderbilt University Institutional Review Board, and all individuals provided written informed consent. Data were collected from April to May 2018, and participants were compensated with a $30 gift card upon completion of the study.

**Fig. 1:**
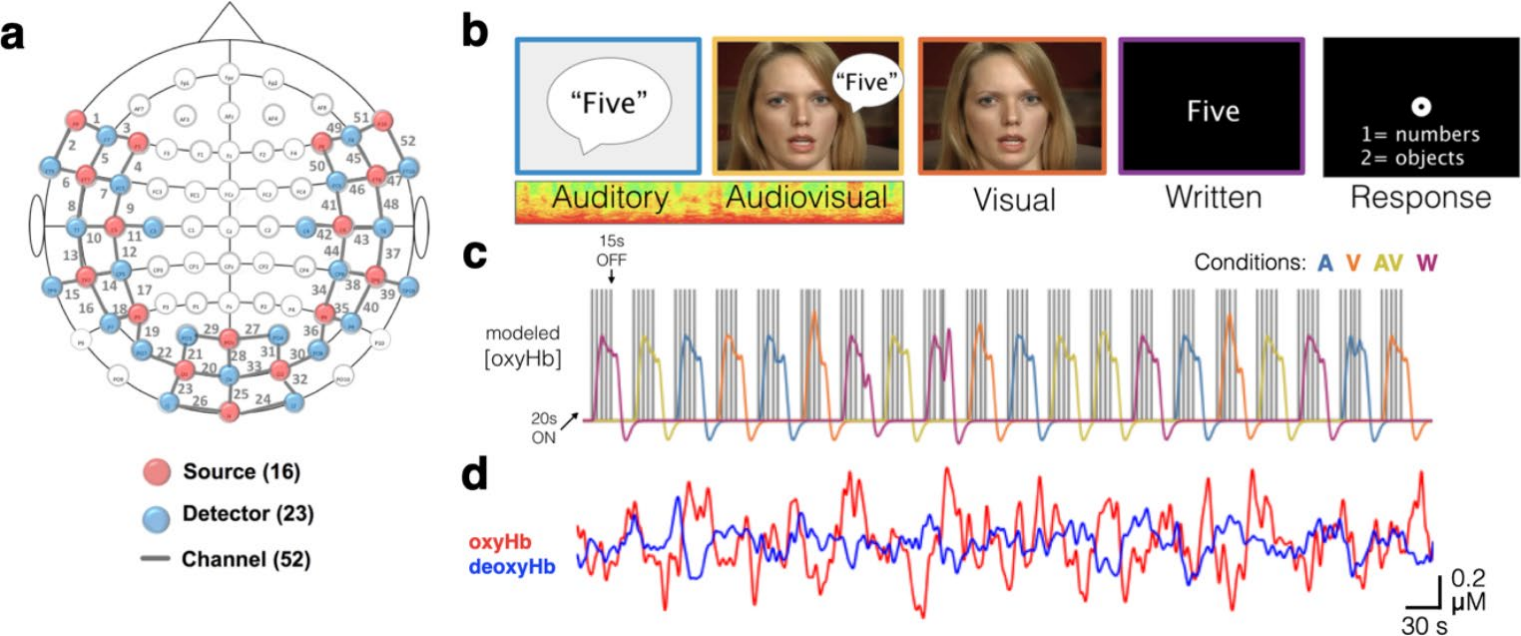
**fNIRS task overview.** A cap containing 16 LED sources and 23 detectors created 52 recording channels (a). During each run, participants were presented with words in four conditions (b) from one of two categories (i.e., numbers v. objects or actions v. animals). Five words were presented in each 20 s block, after which the hemodynamic response function was allowed to return to baseline in a 15s block of silence (c). Following preprocessing, a representative time course for one (of the two 12 min) runs is shown in (d) for a single participant

**fNIRS.** Neuroimaging was completed using NIRScout equipment from NIRx Medical Technologies. 16 LED sources emitting light at 760 and 850 nm wavelengths were surrounded by 23 avalanche photodiode detectors aligned with the 10–20 points shown in Fig. 1a. This arrangement resulted in 52 recording channels with a standard interoptode spacing of 3 cm. Consistent probe placements were achieved by taking measurements for each individual from nasion to inion and between the two preauricular points. From these measurements, we identified Cz that we aligned with the corresponding location on a mesh 10–20 cap containing all optodes. Data were collected in an interleaved manner at a sampling rate of 7.8125 Hz.

The concurrent task involved word categorization in 4 different conditions: auditory-only listening in noise, AV listening in noise, visual-only lipreading, and reading written words (Fig. 1b). Participants listened for a monosyllabic target word, presented at 60 dB SPL, which for the auditory-only and audiovisual conditions, was temporally centered within 2.5s of 4-talker babble. Stimuli were presented via E prime 2.0 software and triggers were sent over a parallel port to mark the beginning of each trial. Participants responded with a keypad to indicate in which of two categories a word belonged (objects v. numbers; actions v. animals; see Supplementary Table S1 for full word lists). Each twenty-word list was displayed at the start of the run along with a practice trial. While the listening component of this task had a similar difficulty compared to behavioral word recognition testing (described below), we made modifications to the fNIRS task that translated to: higher scoring (by providing a closed word list), measurable chance performance (at 50% on account of the two possible categories), and little movement (since responses only required a single key press and not repeating words aloud). These modifications had the advantage of confirming attention through performance such that low scores were more likely explained by individuals either not paying attention or falling asleep rather than having difficulty hearing (as in word recognition testing). The disadvantage is that performance is not equated between tasks.

Each fNIRS trial was convolved with hemodynamic response functions to create a model of the change in oxygenated hemoglobin (oxyHb) concentration (Fig. 1c). Data from two runs were averaged together in the analysis. Speech stimuli were presented at 60 dB SPL and noise levels were calibrated to 66 and 69 dB SPL for −6 and −9 dB SNRs, respectively. Because these can be challenging SNRs, we also included a written condition where participants read the words on the screen. This condition served as a positive control for participants understanding the task in the event that anyone scored at chance in the other conditions. Because the neuroimaging results of the written condition are tangential to our primary aims, their results are included as a supplementary figure (Fig. S1).

Subsequent data processing was done using MNE-Python (Gramfort 2013) using the following steps. Noisy channels with poor scalp contact were eliminated based on a scalp coupling index (SCI) < 0.25 (Pollonini et al. 2014), which resulted in pruning an average of 12 (of 52) channels per participant. Raw intensities were then converted to optical density values that were corrected for motion artifacts using temporal derivative distribution repair [(Fishburn et al. 2019) see Supplementary Fig. S2]. Cardiac and pulmonary artifacts were removed from the data using a zero-phase FIR filter (scipy.signal.firwin with Hamming window) from 0.02 to 0.2 Hz with 0.02 Hz transition bands (SciPy 1.0 Contributors et al. 2020). We computed concentration changes with a modified Beer Lambert law, and averaged all of these fluctuations over the stimulus presentation blocks. The resulting signal changes (Fig. 1d) for each channel were analyzed with an event-based general linear model (GLM) using ordinary least squares (OLS) implemented in the nilearn package (Abraham et al. 2014). The modeled signal described above and shown in Fig. 1c was entered into this regression. Four resulting beta (β) weights (one per condition) were then analyzed across the group for significant activity using linear mixed-effects regression. The interaction of channel and condition was used to predict beta values via statsmodels using the formula “beta ~ -1 + channel:condition,” which includes a random intercept term for subject number (Seabold and Perktold 2010). For additional details, see (Lindstrom and Bates 1988). Significance of model coefficients was inferred using Wald Z tests, and the 208 resulting p-values (52 channels and 4 conditions) were corrected using false-discovery rate (FDR) (Benjamini and Hochberg 1995; Genovese et al. 2002).

Finally, to visualize fNIRS results, we projected Z scores onto the cortical surface of the standard “fsaverage” subject from FreeSurfer, which is aligned to MNI space (Fischl 2012) using MNE-NIRS. Together with the Brodmann atlas (Rorden and Brett 2000), we determined the corresponding anatomical labels using the fOLD toolbox (Zimeo Morais et al. 2018) from https://github.com/nirx/fOLD-public. Because the source/detector pair located at the 10–20 points P5 and PO7 was not available via the fOLD toolbox, we estimated the neighboring location (i.e., P7 in place of P5). Though this estimation is largely overlapping the cortical area of this channel, the reported anatomical specificity values for channel 19 may be slightly inferior to the true values.

The MNE-Python code used for the aforementioned preprocessing and analysis can be accessed on a Github repository via https://github.com/LABSN-pubs/2022-BrainTopogr-fNIRS-AV. All de-identified data and stimuli from this study are also available from the corresponding author upon reasonable request.

**Fig. 2:**
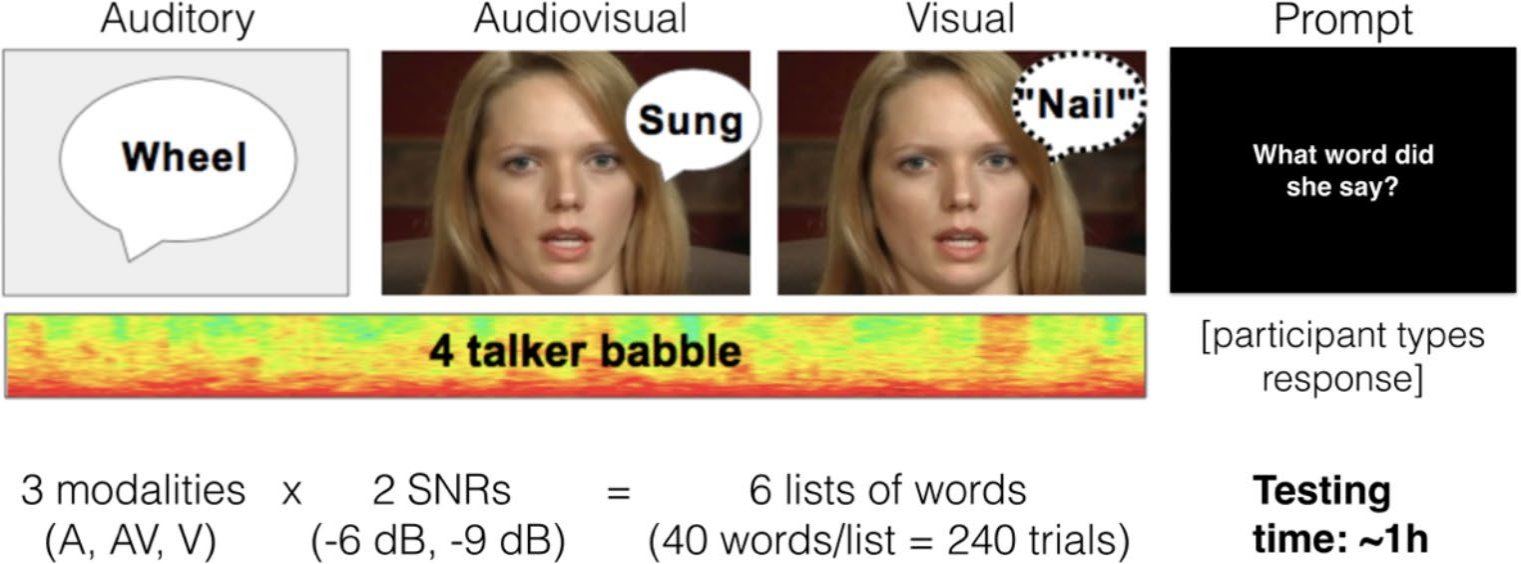
**Word recognition task overview**. For a behavioral correlate of audiovisual speech integration, participants were tested for monosyllabic word recognition in three modalities and two SNRs

**Word recognition in noise.** For a behavioral measure of integrative speech ability, we designed a word recognition task with background noise at two SNRs aimed at achieving speech perception performance in the range typically exhibited by adult CI users in quiet (e.g., Gifford et al. 2018). In lieu of simulating a CI via speech vocoding, we tested a familiar circumstance of listening in the background noise of competing talkers in order to quantify an ecological measure of AV integration. Participants listened for a monosyllabic target word, presented at 60 dB SPL, which was temporally centered within 2.5s of 4-talker babble at either 66 or 69 dB SPL (i.e., −6 and −9 dB SNRs). We calibrated the relative sound levels of the words and noise using a Larson Davis sound level meter and audio files of the long-term average spectrum (LTASS) for each (Donley, Jacob 2017; Donley et al. 2018). Recordings of target words were created by Picou and colleagues who also did intelligibility balancing (Supplementary Fig. S3) to ensure that word lists were well matched for listening difficulty (Picou et al. 2011). In total, we tested 6 lists of 40 words each (Fig. 2; see Supplementary Table S2 for all word lists). We quantified the percent of words correctly identified in each condition, and interactive index (ii) was calculated as a measure of audiovisual gain using the formula: ii = [ AV- max_(A,V)_ ] / AV x 100%. Results were analyzed in R using resampling based on a Welch’s t-test in order to account for different sample distributions [as in (Butera et al. In Press)].

**Fig. 3:**
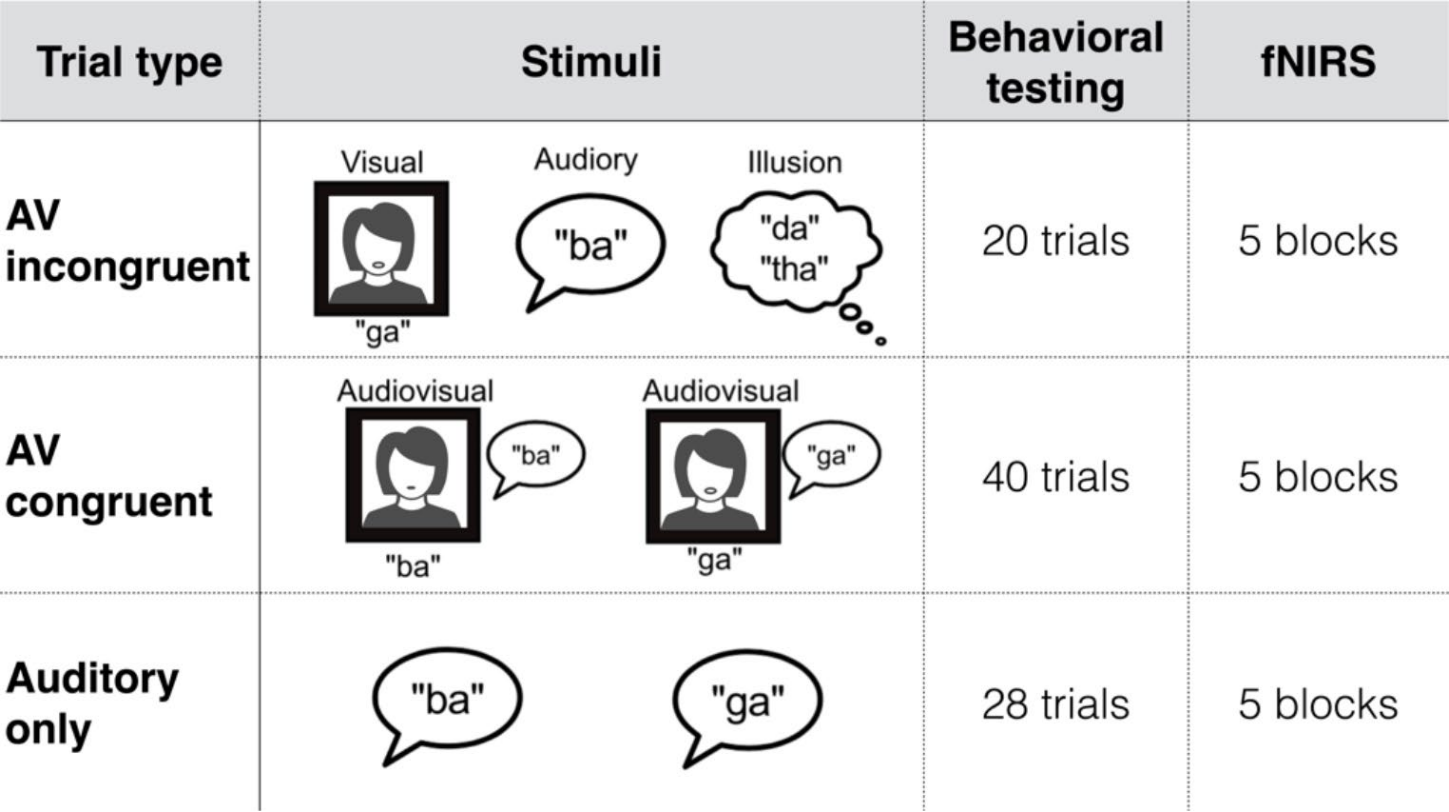
**McGurk experiment overview.** Four trial types in behavioral experiments tested unisensory and audiovisual syllable identification as well as the illusory perception of the novel syllables “da” or “tha”. One fNIRS run measured cortical activity during passive listening to the auditory and AV conditions

**McGurk effect.** We compared behavioral and neuroimaging measures of AV speech integration to perception of the McGurk illusion in this cohort. We measured perception of unisensory control conditions (i.e., auditory-only and visual-only syllables) as well as congruent AV trials in two separate blocks for a total of 88 trials (Fig. 3).

Similar to prior studies using these stimuli Woynaroski et al. 2013; Stevenson et al. 2014c; Butera et al. In Press), videos of a female speaker articulating the syllables “ba” and “ga” were displayed on a CRT monitor approximately 65 cm from participants using Matlab 2008a (Mathworks) and Psychophysics toolbox extensions (Brainard 1997). Auditory stimuli were delivered at a comfortably loud level (approximately 65 dB SPL) through a mono speaker. Participants responded to the question, “What did you hear?” using a keypad with the 4 options: “ba,” “ga,” “da,” or “tha.” Probability of perceiving the illusion was defined as both “da” and “tha” responses, henceforth referred to as “da.” We calculated the probability of perceiving the illusion [p(McGurk)] using a formula that subtracts incorrect “da” responses in unisensory trials from “da” responses to AV McGurk trials: p(McGurk| “da”) x [1 – p(Unisensory|”da”)].

For the fNIRS McGurk task, participants passively listened to three different conditions: incongruent AV stimuli (AVi), congruent AV stimuli (AVc), and auditory-only syllables (Fig. 3). These data were analyzed in the same manner as the fNIRS word categorization task.

Due to skewness in several measures, c﻿orrelations between neuroimaging and behavior were assessed via Kendall’s tau.

## Results

**Fig. 4:**
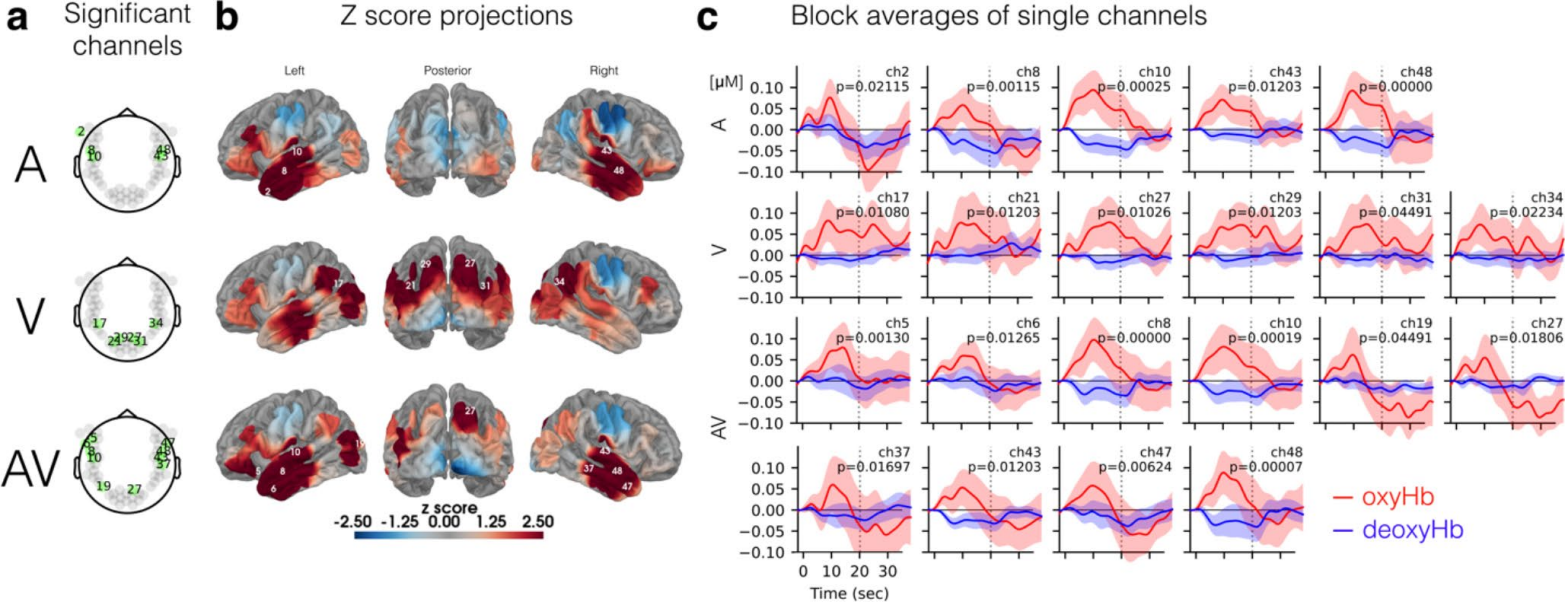
**Cortical activity during the AV word categorization task.** Signal changes in oxygenated hemoglobin were analyzed with an event-based general linear model using ordinary least squares. (a) The resulting β weights for each condition were analyzed for significant activity using a mixed-level model and we found 16 significant (FDR corrected) channels (green highlighted) across all conditions. (b) The higher Z scores of these channels are plotted as warmer colors using heat maps projected onto the pial surface of a standard brain atlas. (c) Block averages of hemoglobin concentration changes for these 16 channels are plotted for oxyHb (red) and deoxyHb (blue) with shaded regions corresponding to 95% confidence intervals for the group

**fNIRS imaging during word categorization.** During fNIRS recordings, participants scored well above chance (i.e., 50%) on the word categorization task, suggesting that they were actively attending to the stimuli for each of the conditions throughout the experiment (Fig. S4). Of the 52 fNIRS recording channels (Fig. 1a), there was significant FDR-corrected activity in 16 channels across the auditory, visual, and AV conditions (Fig. 4).

Five channels had significant activity during the auditory-only condition (Fig. 4). These included bilateral activations of the middle and superior temporal gyrus (MTG and STG) as well as left-lateralized activity in both the inferior temporal gyrus (ITG) and the temporopolar area (Table 1). Block averages of hemoglobin concentration changes for each of these channels display a typical increase in oxyHb concentration that returns to baseline shortly after the stimulus ends (vertical dashed lines in Fig. 4c insets).

Six channels in the visual-only lipreading condition had significant activity (Fig. 4b). All significant channels were bilaterally active across association regions of visual cortex in addition to more anterior regions in the inferior parietal lobule likely to be responsive to visual motion via the dorsal visual processing stream. None of these channels were collocated with those from the auditory-only condition (Fig. 4a), though MTG was active in both conditions. Additional structures underlying these visually-activated channels include the visual cortex (V1), visual association cortices (V2 and V3), STG, the fusiform gyrus, and the angular gyrus (Table 1).

**Table 1:** Significant fNIRS channels during the word categorization task. In total, 16 channels had significant activity during the auditory, visual, and AV word presentation conditions. The Brodmann areas and anatomical landmarks are listed for all specificities over 10%, and because hemispheric specificity differed by 1–5%, values were averaged for contralateral pairs

Channel(s)	Significant condition(s)	Anatomical Landmark	Specificity (%)
2	Auditory	38 - Temporopolar area	42
		20 - Inferior Temporal Gyrus	32
		21 - Middle Temporal Gyrus	26
5	Audiovisual	38 - Temporopolar area	32
		45 - pars triangularis, Broca’s area	24
		48 - Retrosubicular area	16
6, 47	Audiovisual	21 - Middle Temporal Gyrus	68
		38 - Temporopolar area	20
8, 48	Auditory & Audiovisual	21 - Middle Temporal Gyrus	83
10, 43	Auditory & Audiovisual	22 - Superior Temporal Gyrus	45
		21 - Middle Temporal Gyrus	38
17, 34	Visual	39 - Angular gyrus, part of Wernicke’s area	34
		22 - Superior Temporal Gyrus	25
		37 - Fusiform gyrus	17
		21 - Middle Temporal Gyrus	12
19	Audiovisual	19 - Visual Association Cortex (V3)	64
		37 - Fusiform gyrus	32
21, 31	Visual	18 - Visual Association Cortex (V2)	48
		17 - Primary Visual Cortex (V1)	43
27, 29	Visual & Audiovisual	18 - Visual Association Cortex (V2)	66
		19 - Visual Association Cortex (V3)	17
		17 - Primary Visual Cortex (V1)	13
37	Audiovisual	21 - Middle Temporal Gyrus	55
		20 - Inferior Temporal Gyrus	42

There were 10 active channels in the audiovisual condition: 5 in each hemisphere. We saw bilateral activity across the superior and middle temporal gyri as well as visual association cortex (V3), and the temporopolar area. We identified left-lateralized activity the left fusiform gyrus, the retrosubicular area, and the pars triangularis, part of Broca’s area. Finally, we also found right-lateralized activity in the inferior temporal gyrus (IFG) and the right primary and visual association cortices (V1 and V2). Many of these areas, particularly in the left hemisphere, have known responsivity to semantic language tasks including separating aural language from noise (Bishop and Miller 2009). Of the 10 active channels in the audiovisual listening condition, four were also active during auditory-only listening and one was active during visual-only lipreading (Fig. 4).

To test whether there was any greater AV-evoked activity than A-only-evoked activity, we then analyzed contrasts of these conditions with paired t-tests and identified the following channels in the occipital lobe with seemingly greater activity: Ch21 (t_(21)_ = 2.14, p = 0.046), Ch27 (t_(20)_ = 2.84, p = 0.010), and Ch34 (t_(23)_ = 2.33, p = 0.031). All three of these channels were also identified in the visual-only condition as having significant oxyHb concentrations over baseline levels while lipreading. Even so, after corrections for multiple comparisons, none of these three channels met the significance threshold suggesting that the AV speech stimuli did not significantly elicit more activity than auditory stimuli alone for either unisensory or multisensory-selective areas.

**Fig. 5:**
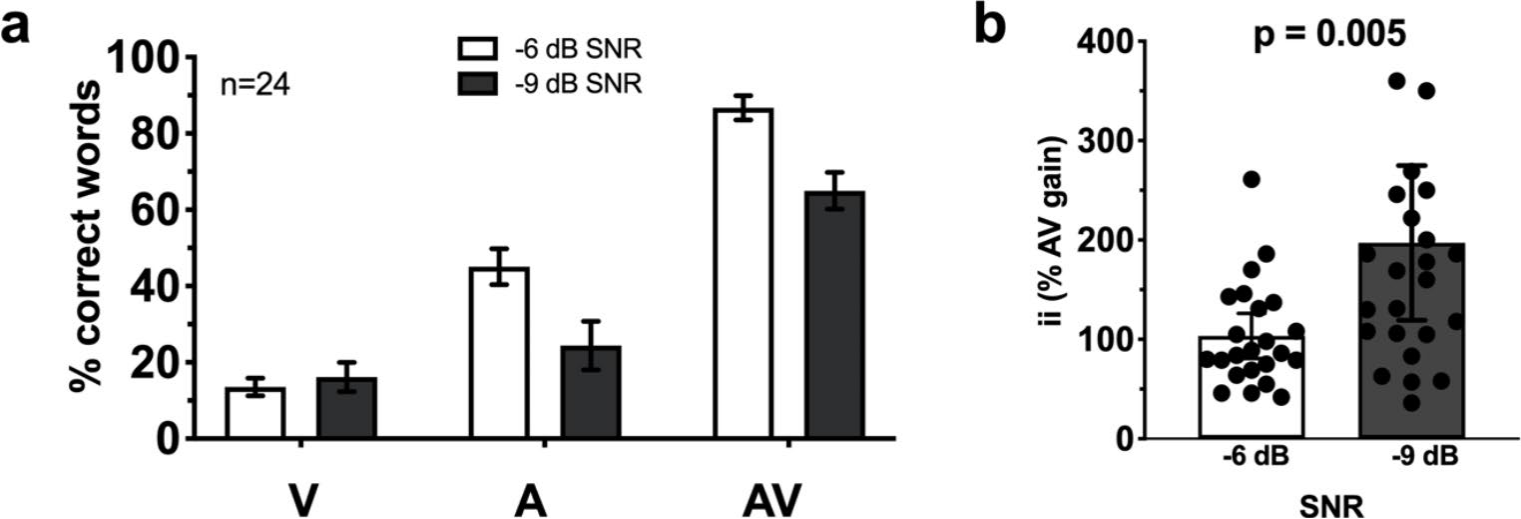
**Audiovisual word recognition results.** Error bars indicate 95% confidence intervals of the mean for the words identified in each list (a) and the interactive index for each SNR (b). Note that for better visualization one ii data point at 960% in the −9 dB SNR condition is omitted from panel (b)

**Behavior in the word recognition task.** In the word recognition task, lipreading (i.e., visual) performance was similar between the two noise levels (Fig. 5a). Group means were 14% correct for the −6 dB SNR condition and 16% correct for the −9 dB SNR condition. During auditory-only listening, mean performance was 45% correct in the −6 dB SNR condition and 24% correct in the −9 dB SNR condition. A simple addition of the performance in these auditory and visual conditions would result in predicted performance of 58% and 41% correct. However, actual performance was at 87% and 65% for the −9 dB SNRs, respectively, illustrating a superadditive gain under AV listening conditions. The magnitude of AV gain (represented by ii; Fig. 5b) was significantly higher in the louder noise level we tested (t_(25.4)_ = -2.3, p_adj_ = 0.005) indicating greater AV benefit in this more difficult listening condition (103% v. 197%).

**Fig. 6:**
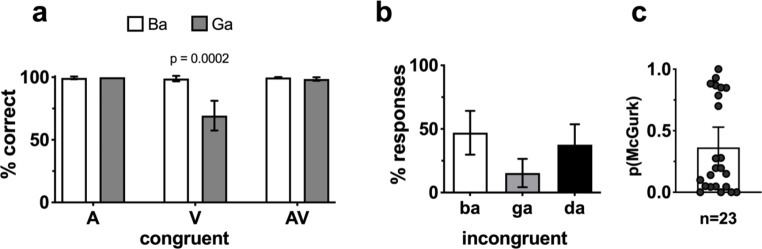
**McGurk behavioral task results.** Control trials of unisensory and congruent AV syllable identification were at or near ceiling with the exception of lipreading “ga” (a). Responses to the McGurk trials were distributed among the auditory token “ba”, followed by the fused token, and lastly, the visual token “ga” (b). The probability of perceiving the illusion has a large, somewhat bimodal distribution (c)

**McGurk tasks.** In the control trials, there was nearly identical at-ceiling performance for auditory-only and audiovisual identification of “ba” and “ga” (Fig. 6a). However, there was significantly lower lipreading performance for the visual “ga” when compared with the bilabial articulation of “ba” (t_(23)_ = 4.46, p = 0.0002). The McGurk trials elicited responses for each component stimulus (i.e., auditory “ba” and visual “ga”) as well as for the fused syllable (“da”) (Fig. 6b). The average probability of perceiving the illusion was 0.36, though there was enormous inter-individual variability in the reporting of the fused token that is better illustrated in the distribution of individual data than the group average (Fig. 6c).

**Fig. 7:**
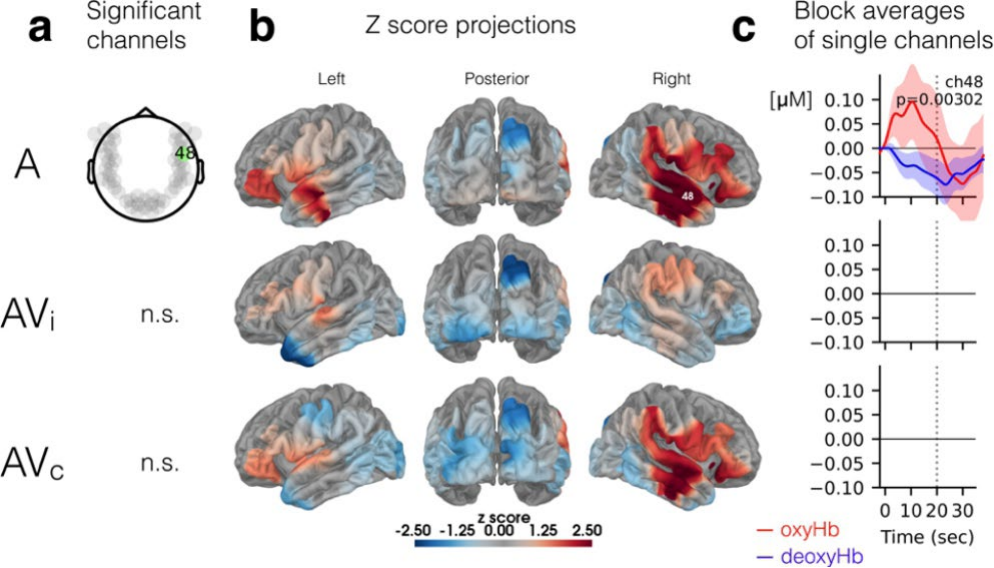
**Cortical activity in response to auditory alone (A), incongruent AV (i.e., AVi or McGurk) and congruent AV stimuli (AVc).** Significant (FDR corrected) channel (a), Z score projections (b), and block averages (c) are shown for the auditory-only listening condition, incongruent AV stimuli, and congruent AV stimuli

In the fNIRS task with passive listening to McGurk stimuli, there was only one significant channel for the Auditory-only condition (Fig. 7a). Located over the middle temporal cortex, this channel was also active in the word recognition task during A and AV conditions (Fig. 4). There were no significant channels for either the AVc or for the AVi stimuli in which individuals may experience the McGurk effect.

**Correlations with behavior.** Across all conditions, we found activity in three channels that initially correlated with the behavioral measures of p(McGurk) and the magnitude of multisensory integration (i.e., ii). In the visual condition of the word recognition task, channel 17 had an uncorrected correlation with ii from the −9 dB SNR testing (τ= -0.30, p = 0.04), and this channel correlated with p(McGurk) as well (τ= 0.41, p = 0.01). Also in the visual condition, channels 29 and 34 had an uncorrected correlation with p(McGurk) of τ= 0.33, p = 0.03 and τ= 0.35, p = 0.04, respectively. No fNIRS channels correlated with behavioral measures of p(McGurk) or ii after correcting for multiple comparisons.

### DISCUSSION

In this study we designed two AV speech-in-noise tasks, one for deriving a behavioral measure of multisensory integration (i.e., interactive index) and one for measuring cortical hemodynamics using functional near infrared spectroscopy (fNIRS). To best simulate everyday circumstances of multisensory integration, testing was done in the presence of multi-talker background noise in two difficult-to-hear SNRs. In behavioral testing, we found that background noise that was 6 and 9 decibels louder than monosyllabic target words resulted in averages of 45% and 24% of words correctly identified in auditory-only listening (Fig. 5a). These values approximate reasonable CI-like performance ranges in quiet (Blamey et al. 2013b; Duchesne et al. 2017) and are sufficiently low enough for the addition of visual articulations to result in multisensory gain (Ross et al. 2007). Indeed, adding visuals prompted superadditive gain of 103% and 197% improvements over auditory alone conditions (Fig. 5b). The significant difference between these measures illustrates how increasing levels of background noise reduces auditory saliency, and increases audiovisual benefit—a process termed inverse effectiveness (Holmes 2007). Having confirmed significant audiovisual benefit in this behavioral task, we then investigated the cortical hemodynamics in three different listening modalities.

We identified significant, bilateral activity for both auditory-only and AV listening conditions in the middle and superior temporal cortex (Fig. 4). This finding satisfies our primary goal for identifying known auditory and language networks using a speech-in-noise task and optical imaging. More specifically, compared to the auditory-only condition, we saw additional AV-evoked responses in the left fusiform gyrus, Broca’s area, the left retrosubicular area, right visual areas (V1-2) and bilaterally in visual association cortex (V3). This activation pattern suggests broad, partly left-lateralized activity involving: phonemic and semantic language-processing networks, visuospatial processing of facial movements, and one of the major hubs in the brain for the convergence of auditory and visual information—the superior temporal cortex (Stevenson et al. 2007).

In particular, the superior temporal sulcus (STS) receives convergent input from both auditory association areas and extrastriate visual areas, and has been implicated in both AV speech integration as well as perception of the McGurk illusion (Beauchamp et al. 2004, 2010). Anatomically, the STS separates the superior and middle temporal gyri, starting at the temporal pole and terminating at the angular gyrus of the inferior parietal lobe, making it the second longest sulcus in the brain. While posterior regions of STS are responsive to visual integration, middle regions respond more broadly to biological motion, and the mid-posterior regions in between are particularly responsive to moving faces and voices (Deen et al. 2015). The optodes in our fNIRS montage were spaced a standard distance of 3 cm, which affords an optimal path length for infrared light to transmit up to 15 mm into cortical tissue before scattering (Strangman et al. 2014). This physical limitation to infrared spectroscopy means that each recording channel created by neighboring pairs of optodes covers both a broad and shallow region of cortex. As a result, we cannot differentiate activity between neighboring structures like the superior temporal gyrus versus the sulcus. However, we did see elevated activity bilaterally in several channels and in reoccurring patterns between similar conditions (e.g., 40% of AV-responsive channels were also active during auditory-only listening). Thus, we found redundant support for the involvement of several key areas of the temporal lobe in both unisensory and multisensory speech processing.

Because of the large number of recording channels in this study, we had to correct for multiple comparisons (i.e., 208) to control type I error. For simplicity, we used false discovery rate, which does not consider the spatial correlations that are present in the sensor data (Benjamini and Hochberg 1995), and as such is likely too conservative (i.e., inflates type II error). Our goal, however, with the current fNIRS acquisition methodology (adhering to 10–20 locations) and statistical testing (i.e., within-subject tests across a given condition) was to focus on finding consistent channel activation patterns across subjects. This approach assumes consistent probe placement between subjects and has its advantages and disadvantages. One disadvantage is that it fails to capture individual spatial variability in neural activation. One advantage is that successfully identifying significant effects (after multiple-comparisons correction) allowed us to then infer brain-region-specific effects at the population level using virtual co-registration with structural databases (Zimeo Morais et al. 2018). An alternative approach of an ROI-based analysis would be a valuable follow-up study where, for example, separate conditions—independent of the conditions of interest—are included to establish functional ROIs. Additional technological improvements could include using a 3D digitizer to record the registration of individual optodes for greater corresponding placements of channels between subjects. Additionally, including short channel recordings would enable regressing out extracerebral signal changes (Goodwin et al. 2014). That is, in lieu of the preprocessing described here, this analytic approach enters systemic changes into a GLM to better differentiate functional brain activity from absorption changes due to respiration, pulse, and other physiological fluctuations in the scalp and cerebrospinal fluid. This methodological change could increase the likelihood of detecting more subtle effects. Along the same lines, more dense recordings with many overlapping channel distances can allow for a more detailed understanding of activity in a small area of interest (Pollonini et al. 2014; Hassanpour et al. 2015; Olds et al. 2016). Thus, the broad coverage of the 52 optodes in this study (Fig. 1a) had the benefit of visualizing bilateral activity across all four lobes of the brain, but this also led to an overly conservative criterion for statistical significance, which may be underestimating the extent of neural activations elicited by these tasks.

This may be particularly true for the McGurk task where we identified just one significant channel in the right middle temporal cortex during the auditory-only listening condition and none during AV conditions (Fig. 7). In the only other fNIRS study of the McGurk effect that we’re aware of, the authors identified left temporal lobe activation to congruent AV stimuli as well as bilateral temporal activity to incongruent stimuli, presumably during the McGurk illusion (Ujiie et al. 2020). Given that the present study tests adults, whereas Ujiie and colleagues (2020) tested 8–9 month old infants, it is difficult to compare results. Besides obvious developmental differences, infrared light can travel several centimeters into the cortex of infants, which means that functional neuroimaging can probe activity at much greater depths in infants compared to adults (Issard and Gervain 2018). As a result, fNIRS in adults is less likely to include activity from sulci than it is in infants. However, transcranial magnetic stimulation (TMS) in the vicinity of left STS has been shown to disrupt perception of the McGurk illusion in adults (Beauchamp et al. 2010), and this finding was determined using equipment with a half-value depth (d_1/2_) of 1.5 cm (Deng et al. 2013). This means the electrical field strength is one half its maximum value at a depth of 1.5 cm below the cortical surface; therefore, a causal disruption of the McGurk effect by TMS occurred over an area of temporal cortex that reached a similar, **albeit maximum**, depth to what we probed via fNIRS (Scholkmann et al. 2014). In order to better replicate the fMRI literature (Nath et al. 2011; Nath and Beauchamp 2012), further studies are needed to characterize superficial, optically-derived activation patterns of McGurk-evoked activity—perhaps focusing on high-density optical imaging methods to maximize penetration depth beyond 2 cm (Dehghani et al. 2009) and better reach deeper structures like STS.

Though inverse effectiveness has been well documented at the level of individual neurons (Stein et al. 2014), superadditive integration is less clearly seen in hemodynamic signals like the oxyHb concentrations upon which we modeled activity (Fig. 4c). Nevertheless, we contrasted the A and AV listening conditions. We found only preliminary differences in neural activity for three channels that were also identified in the lipreading condition. Thus, we did not see the audiovisual condition elicit greater recruitment of auditory-responsive channels as some have shown using fMRI (Erickson et al. 2014). Instead, this contrast identified the added involvement of visual channels when listening audiovisually. Although the −9 dB SNR resulted in relatively low auditory-only identification of just 24% of words on average, even greater noise levels would presumably increase integration further and may be necessary to fully explore the possibility of superadditive AV channels. Thus, in future studies, it may be beneficial to add higher competing background noise (i.e., for a lower, more difficult SNR) in order to potentially elevate oxyHb concentrations beyond what we report here and to make this contrast between AV and A-only conditions more pronounced (Callan et al. 2003; Stevenson and James 2009).

Silent lipreading recruited bilateral activity in visual association areas as well as more anterior channels likely to be associated with motion perception (Fig. 4a). In particular, we saw activity in inferior parietal channels that aligns well with the dorsal visual processing stream. Though traditionally associated with orthographic reading, some studies also suggest broader responsivity in the visual word form area (VWFA) to include lipreading (Chen et al. 2019). We identified activity bilaterally that encompasses this area in the left fusiform gyrus (i.e., ch 17 and 19). Additionally, visually-evoked activity in the left superior temporal cortex may correspond to a biological motion-responsive region with inputs to adjacent multisensory areas also in the STC (Pitcher and Ungerleider 2021).

**The McGurk effect** has been widely tested for some time (McGurk and MacDonald 1976), though some studies highlight the disparate mechanisms behind this illusion and integration of natural, congruent speech [e.g., (Van Engen et al. 2017)]. In behavioral testing, we found substantially lower success in lipreading the syllable “ga” compared to “ba,” which highlights their differences in visual ambiguity that are key for this illusion. While fusion rates vary widely across different stimuli, the average probability of perceiving the McGurk illusion in this study was 0.36. This value is both consistent with prior studies using this particular stimulus Woynaroski et al. 2013; Stevenson et al. 2014c; Butera et al. In Press) and is well within typical ranges for other stimuli [i.e., fusion varied between 0.17 and 0.81 across a 14 stimulus set; (Magnotti and Beauchamp 2015)]. Recent work in modeling the cue combination that occurs in the McGurk illusion returns subject-specific parameters like sensory noise and threshold that, unlike our p(McGurk) results are stimulus independent (Magnotti and Beauchamp 2015; Stropahl et al. 2017). Because these methods require testing multiple McGurk stimuli of varying strengths to find individual-level thresholds, they are not applicable to the present study but would be an interesting follow up.

The somewhat bimodal distribution in illusion percepts has also been noted previously and likened to an “all-or-nothing” effect with the majority of individuals falling at the extremes (Fig. 6c). Though typical for this task, this distribution likely presented a confound in our fNIRS analysis of the McGurk stimuli. Specifically, further segmentation of the group into illusion perceivers and non-perceivers may provide more insight on differing activation patterns. Here, however, we report only group-level effects with all 23 participants, since any further analyses are likely to be underpowered, which is a broader issue in the McGurk literature that has been highlighted recently (Magnotti et al. 2020). Thus, the one channel that was active in the right middle temporal cortex for the auditory-only condition was determined in an analysis of all subjects together (Fig. 7). This channel was also active during the A and AV conditions of the word recognition task, which further supports its role in both auditory and audiovisual processing. It is important to point out that the McGurk stimuli were delivered passively such that participants were not forced to report their perception. As a result, we cannot compare these results to active listening, which is known to influence both the magnitude and area of BOLD signal changes (Binder et al. 2008). Hence, an active task could make this contrast more robust, identifying otherwise subthreshold activity in the left hemisphere. Furthermore, given the relatively low incidence of McGurk percepts across all participants, structuring the task (or perhaps instating selective recruitment of high and low perceivers) could be more effective in identifying areas likely to be involved in McGurk perception using fNIRS.

Relevant for future studies in CI users, crossmodal plasticity may also play a role in facilitating the McGurk effect (Stropahl and Debener 2017). In a study using EEG source localization, the authors report the crossmodal activation of auditory cortex in CI users in response to faces, which had a positive relationship with the degree of McGurk illusion perception. Interestingly, a similar effect was also seen with a moderately hearing-impaired group, likely suggesting an early onset of these adaptive changes in CI users and supporting further inquiry into this task using fNIRS.

To relate the neuroimaging and behavioral results in this study, we tested for correlations between ii and p(McGurk) with the activity across all channels and conditions. We found three channels from the visual condition that had positive correlations with p(McGurk): channel 29 located in the right primary and visual associative cortex, and bilateral activity in channels 17 and 34 in the temporal and inferior parietal lobule and likely playing a role in the extrastriate visual processing of the dorsal “where” stream. This finding suggests that increased perception of the McGurk illusion is also associated with greater cortical recruitment of these visual motion processing channels while lipreading, and prior studies have suggested lipreading skill as a contributing factor for individual differences in McGurk perception (Strand et al. 2014). Curiously, despite the high similarity between the fNIRS speech-in-noise task and the behavioral word recognition in noise task, the only correlation we found between these experiments was a negative relationship between visually-evoked activity in channel 17 and ii. This suggests that individuals who experienced greater audiovisual gain also had lower evoked activity in this visual-motion responsive channel. Because the brain-behavior correlations in this study fail to meet statistical thresholds after correcting for multiple comparisons, it is possible that the uncorrected correlations that we report are spurious, and therefore, require further investigation.

Elsewhere in the literature, fNIRS is proving to be a particularly suitable technique for carrying out brain imaging in CI users (Saliba et al. 2016). A growing body of work supports the sensitivity of fNIRS for measuring speech-evoked activity in normal-hearing (NH) controls (Pollonini et al. 2014; Defenderfer et al. 2017) and CI users (Sevy et al. 2010). Furthermore, fNIRS has been used to distinguish activity patterns between proficient and non-proficient CI users (Olds et al. 2015), to map unique phonological awareness networks in non-proficient CI users (Bisconti et al. 2016), and to measure multisensory interactions for both non-speech (Wiggins and Hartley 2015) and speech stimuli (van de Rijt et al. 2016; Anderson et al. 2017). Adding to this body of literature, the present study reports behavioral performance in a normal hearing cohort for both the McGurk effect and speech-in-noise with broad neural activations evident during auditory-only listening, visual-only lipreading, and audiovisual listening. Future studies assessing these tasks in CI users will enable comparisons of how the magnitude and mechanisms of audiovisual speech integration may differ for this clinical population.

In conclusion, vision is known to play a critical role in communication, and yet clinical assessments of CI candidacy and longitudinal postoperative outcomes have largely been limited to auditory-only measures. Consequently, current assessments of CI outcomes are unable to describe the comprehensive profile of functional communication. We believe that a thorough investigation of visual abilities is the first step toward better understanding ecological listening proficiency. The present study provides further evidence that fNIRS is a useful tool for investigating brain-behavior connections in the context of a speech-in-noise paradigm that effectively reduces auditory saliency with the addition of background noise and necessitates greater perceptual integration with complementary visual cues. This foundational work in normal hearing controls confirms speech-evoked activity in known auditory and semantic language processing areas along the superior and middle temporal cortex. Furthermore, we show the lateralization of AV-evoked activity toward speech networks on the left, higher-level visual motion processing on the right, and broad lipreading-evoked activity in both the occipital cortex and anterodorsal regions of motion processing. Future work will compare this activity in normal hearing listeners to cochlear implant users, for whom audiovisual integration is a daily necessity and which may be mechanistically carried out in a unique fashion following the potential crossmodal effects of hearing loss and adaptive plasticity.

## Electronic supplementary material

Supplementary Material 1

## Data Availability

De-identified data and stimuli from this study are available from the corresponding author upon reasonable request.

## Code Availability

Python code used in the fNIRS data analysis is available on Github via: https://github.com/LABSN-pubs/2022-BrainTopogr-fNIRS-AV
