# Supplementary material for: Functional localization of audiovisual speech using near infrared spectroscopy
Source: Brain Topogr. 2022 Jul 12;35(4):416–30. doi: 10.1007/s10548-022-00904-1 (PMC9334437; doi:10.1007/s10548-022-00904-1)
Supplement: Supplementary file 1 — Supplementary Material 1 [file 10548_2022_904_MOESM1_ESM.docx]

**Appendices**

|  | fNIRS Run 1 | | fNIRS Run 2 | |
| --- | --- | --- | --- | --- |
|  | Objects | Numbers | Animals | Actions |
| 1 | ball | one | bear | cheer |
| 2 | bed | two | bee | read |
| 3 | bell | three | bird | knock |
| 4 | bike | four | bug | thank |
| 5 | book | five | calf | tell |
| 6 | box | six | cat | wish |
| 7 | chair | seven | crab | chat |
| 8 | clock | eight | dog | ride |
| 9 | cup | nine | duck | run |
| 10 | map | ten | fish | walk |
| 11 | nail | eleven | frog | toss |
| 12 | note | twelve | goose | look |
| 13 | page | thirteen | hen | fold |
| 14 | rag | fourteen | horse | catch |
| 15 | ring | fifteen | moth | press |
| 16 | shirt | sixteen | mouse | guess |
| 17 | shoe | seventeen | rat | teach |
| 18 | spoon | eighteen | sheep | fall |
| 19 | truck | nineteen | snake | take |
| 20 | wheel | twenty | toad | cut |

**Table S1. fNIRS word lists.** Testing included 4 categories of 20 words each for a total of 80 words.

|  | A High SNR | AV High SNR | V High SNR | A Med SNR | AV Med SNR | V Med SNR | A Low SNR | AV Low SNR | V Low SNR |
| --- | --- | --- | --- | --- | --- | --- | --- | --- | --- |
|  | **List 1** | **List 2** | **List 3** | **List 4** | **List 5** | **List 6** | **List 7** | **List 8** | **List 9** |
| 1 | No | Dodge | Shoe | Tar | Sung | Fail | Yam | Which | Match |
| 2 | Look | Nail | Tar | Quick | Raw | Learn | Chat | Snake | Sane |
| 3 | Jug | Tray | Leave | Raid | Loose | Yam | Choose | Kite | Germ |
| 4 | Beg | Week | Gone | Road | Paste | Life | If | Pinch | Web |
| 5 | Shore | Kite | Beet | Cheek | Hush | Patch | Big | Hiss | Soon |
| 6 | Head | Judge | Hall | Neat | Lack | Have | Rug | Kick | Fudge |
| 7 | Meek | Pool | Run | Vine | Haze | Near | Limb | Coat | Mouth |
| 8 | Shawl | Soap | Knock | Thank | For | Burn | Wife | Peak | Rob |
| 9 | Bought | Low | Thank | Doll | Note | Shout | Kick | Ship | Map |
| 10 | Class | Guess | Get | Foot | Hike | Few | Rich | Path | Rough |
| 11 | Long | Wish | Tooth | Cool | Is | Freeze | Bush | Your | Pole |
| 12 | Lean | Chill | Pearl | Bud | Gray | Them | Hole | His | Fan |
| 13 | Tip | Cool | Phone | Puff | Wash | Base | Camp | Sung | Sour |
| 14 | Tell | Fail | Weight | Sob | Tongue | Cut | Sin | Slice | Cab |
| 15 | Nuts | Fit | Juice | Veal | Loud | Beet | Tape | Chore | Led |
| 16 | Beef | Raid | Loaf | Rat | Mop | Pearl | Girl | Wrong | Gum |
| 17 | Birth | Press | Merge | Dig | Rough | Purge | Scab | Clock | Rib |
| 18 | Boat | Cab | Sob | Shock | Pod | Tray | Death | White | Bone |
| 19 | Sun | What | Sour | Fit | Geese | Bean | Path | Set | Nice |
| 20 | Weed | Third | Clown | Gap | Keep | Falls | Sure | Third | Cause |
| 21 | Nail | Those | Hand | Deck | Mouse | Food | Read | Long | End |
| 22 | Dish | Calm | Led | Get | Jay | Doom | Gas | Loop | Such |
| 23 | Noise | Vote | Came | Hike | As | Jade | Else | Make | Late |
| 24 | Cage | Wheel | Own | Love | Said | On | Nice | Need | Roof |
| 25 | Cape | Youth | Laugh | Peg | Red | Thick | Me | Great | Wag |
| 26 | Mess | Void | Half | Page | Search | Loose | Grab | Doom | Peace |
| 27 | Goose | Ton | Home | Mine | Which | Judge | Rat | Move | Watch |
| 28 | Mill | Niece | Purge | Mode | Shack | Nick | Shore | Grew | Sin |
| 29 | Shine | Goal | Set | Weight | Coin | Moon | Meek | Chalk | Perch |
| 30 | Loop | Live (ai) | Most | Tool | Gin | Sag | Nap | Time | Safe |
| 31 | School | Fade | Tick | Raw | Yearn | Bee | Bone | Kid | Cup |
| 32 | Fair | Are | End | Live | Fine | See | Axe | Chief | Team |
| 33 | Sail | White | Slice | Meet | Slip | Move | Dead | Case | Feet |
| 34 | Whip | Life | Sink | Chin | Pants | As | Gale | Blind | Hull |
| 35 | That | Knife | Rain | Wish | Mode | Mood | Ripe | Burn | Teach |
| 36 | Black | Lip | Hit | Train | Jar | Next | Wide | Hair | Press |
| 37 | Hole | Mate | Reach | Rig | Mood | Note | Tire | Ball | Sink |
| 38 | House | Take | Kill | Mop | Deep | Half | Pain | Crab | Nest |
| 39 | Name | Air | Gum | Gore | Hull | Sure | Check | Sack | Hunt |
| 40 | Low | Tool | Jade | Rot | Drop | Cake | South | Next | Waste |

**Table S2. Word recognition word lists.** Testing included 9 word lists with 286 unique words and 360 trials total.


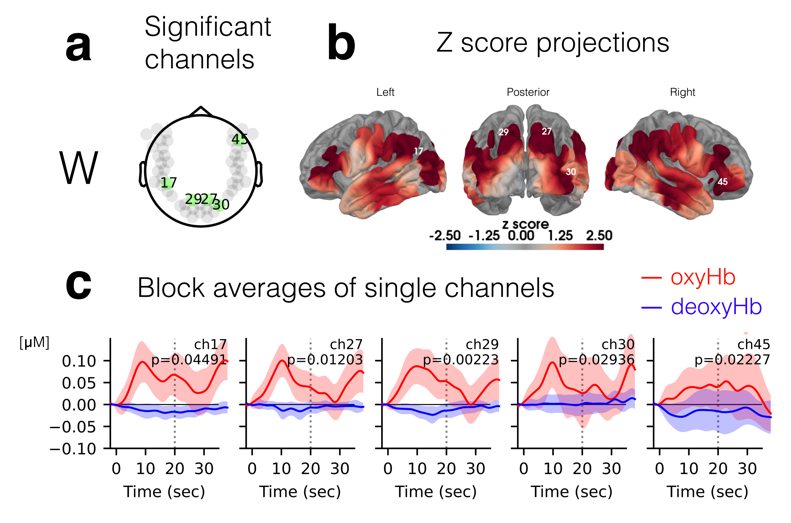


**Figure S1. Cortical activity during Written condition.** Five significant (uncorrected) channels during the written condition (a) are also plotted as warmer colors with higher Z scores within the occipital lobe and broadly across the right hemisphere (b). Block averages of these channels are also plotted with changes in oxyHb (red) and deoxyHb (blue) for the group with 95% confidence intervals as shaded regions (c).


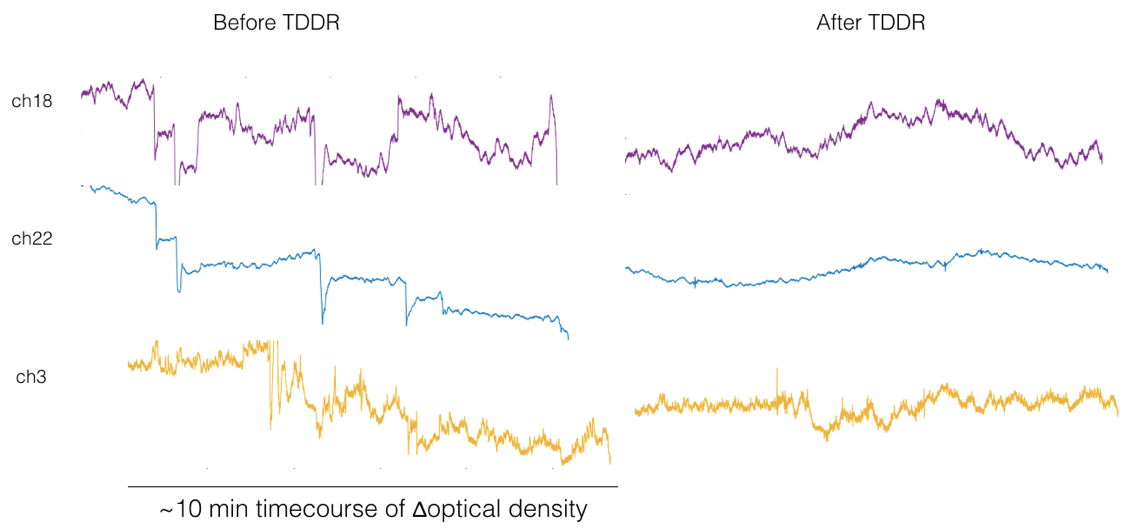


**Figure S2. Example fNIRS filtering with Temporal Derivative Distribution Repair (TDDR).** Three example channels containing motion artifacts were corrected.

**Figure S3. Intelligibility matching word lists.** The 9 word lists for the word recognition testing were matched for intelligibility based on word identification in -3 and -7 dB SNRs by 20 normal-hearing participants as described by Picou et al. (2011).


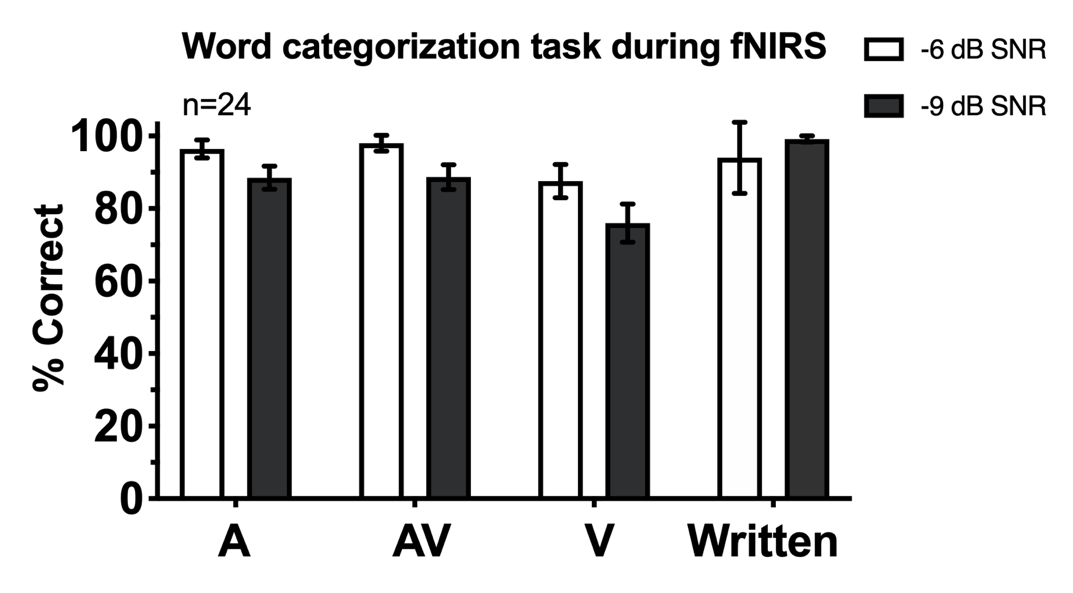


**FIGURE S4. Results for the word categorization task during fNIRS.** This task was designed to maintain attention during fNIRS, and results indicate high accuracy across all conditions for each word group and signal-to-noise ratio (i.e., numbers v. objects for the -6 dB SNR and action verbs v. animals for -9 dB). Chance performance on this task is 50%.
